# Mapping Mobility: Utilizing Local-Knowledge-Derived Activity Space to Estimate Exposure to Ambient Air Pollution among Individuals Experiencing Unsheltered Homelessness

**DOI:** 10.3390/ijerph19105842

**Published:** 2022-05-11

**Authors:** Maeve G. MacMurdo, Karen B. Mulloy, Daniel A. Culver, Charles W. Felix, Andrew J. Curtis, Jayakrishnan Ajayakumar, Jacqueline Curtis

**Affiliations:** 1 Cleveland Clinic Respiratory Institute, Cleveland, OH 44195, USA; culverd@ccf.org; 2 Department of Family Medicine and Community Health, Case Western Reserve University, Cleveland, OH 44106, USA; kbm30@case.edu; 3 Tulare County Counsel, Visalia, CA 93291, USA; cwfelix@tularecounty.ca.gov; 4 Department of Population and Quantitative Health Sciences, Case Western Reserve University, Cleveland, OH 44106, USA; ajc321@case.edu (A.J.C.); jxa421@case.edu (J.A.); jxc1546@case.edu (J.C.)

**Keywords:** homelessness, local knowledge mapping, environmental justice, particulate matter, homeless persons, vulnerable populations

## Abstract

Individuals experiencing homelessness represent a growing population in the United States. Air pollution exposure among individuals experiencing homelessness has not been quantified. Utilizing local knowledge mapping, we generated activity spaces for 62 individuals experiencing homelessness residing in a semi-rural county within the United States. Satellite derived measurements of fine particulate matter (PM2.5) were utilized to estimate annual exposure to air pollution experienced by our participants, as well as differences in the variation in estimated PM2.5 at the local scale compared with stationary monitor data and point location estimates for the same period. Spatial variation in exposure to PM2.5 was detected between participants at both the point and activity space level. Among all participants, annual median PM2.5 exposure was 16.22 μg/m^3^, exceeding the National Air Quality Standard. Local knowledge mapping represents a novel mechanism to capture mobility patterns and investigate exposure to air pollution within vulnerable populations. Reliance on stationary monitor data to estimate air pollution exposure may lead to exposure misclassification, particularly in rural and semirural regions where monitoring is limited.

## 1. Introduction

Increasing data suggest that vulnerable populations may face a disproportionate burden of air pollution exposure. Within the United States, residence in a non-white majority or low income census tract is associated with an increased level of ambient exposure to fine particulate matter [1,2]. Clustering of toxic release inventory (TRI) facilities and other sources of stationary emissions has also been noted within low income and minority communities [3,4]. The patterns of exposure to air pollution experienced by vulnerable populations within these communities remains unknown.

Individuals experiencing unsheltered homelessness make up a growing proportion of the population within the United States [5,6]. In the U.S., over half a million people were documented as experiencing homelessness in 2020 [7,8]. Individuals experiencing unsheltered homelessness are more likely to be chronically homeless, and to experience chronic health conditions [9]. Individuals experiencing unsheltered homelessness are uniquely vulnerable to the impact of worsening air quality, particularly outside of large urban centers where access to indoor shelters may be limited. Additionally, residence in embankments, streets, and structures not designed for human habitation may result in exposure to air pollution from stationary sources that are not well captured by global level analysis.

Few studies to date have attempted to investigate air pollution exposure among individuals experiencing homelessness. In a cross-sectional study of homeless individuals in Utah, self-reported exposure to air pollution was common, with 90% of individuals describing poor air quality [10]. Among this sample, respiratory complaints were common [10]. However, actual exposure to ambient air pollution was not quantified. Exposure to ambient air pollution has been associated with an increased risk of all-cause mortality and cardiovascular and respiratory disease [11,12]. When compared with the general population, individuals experiencing homelessness are already known to be at increased risk of chronic respiratory disease and premature death [9,13,14,15]. While this increased risk of morbidity and mortality is driven by multiple factors, exposure to air pollution may represent an under recognized contributor to negative health outcomes in this population. Quantifying this exposure has significant implications for public health, and also provides important evidence in support of policies aimed at improving access to housing among individuals experiencing unsheltered homelessness.

However, quantifying exposure is not straightforward. A major challenge to assessing air pollution exposure among individuals experiencing homelessness is capturing where participants spend their time in order to measure potential exposures. By definition, there is no home address. The majority of studies assessing environmental air pollution exposure make an assumption of stationarity—that an individual’s residence (or place of employment) represents their only source of exposure [16]. Particularly for individuals experiencing unsheltered homelessness, mobility patterns throughout the day may significantly impact exposure to air pollution. We have previously shown that individuals experiencing homelessness may face exposure to air pollution from multiple sources, including traffic-related air pollution and commercial stationary emitters [17].

To date, the majority of research has focused on the experience of urban homelessness. Rural homelessness is comparatively under-researched. The true amount of people experiencing rural homelessness is unknown, although national survey data estimate that at least 18% of individuals experiencing homelessness reside in rural areas [18]. Rural residents may also face significant exposure to air pollution, as a result of agricultural emissions and roadside traffic [19]. Quantifying rural and semi-rural air pollution also faces several challenges. Air pollution is increasingly recognized to display significant spatial-temporal variation [20]. The majority of stationary monitors are clustered in urban areas [21]. By comparison, many rural sites may lack stationary air pollution monitors. Extrapolating monitor data across regions where monitor sites are limited may lead to under- or over-estimation of true air pollution exposure [22,23]. Rural and semi-rural public health agencies may lack the resources necessary to deploy more traditional approaches to quantifying air pollution exposure.

In our study, we aimed to examine the feasibility of utilizing satellite-derived measures of PM2.5 to quantify exposure to air pollution among individuals experiencing homelessness. Remote sensing data are able to provide an estimate of exposure at a significantly more granular level when compared with stationary monitors, potentially allowing for increased recognition of local “hot spots” that may not be well captured by traditional approaches [24,25]. Satellite-derived data are publicly available and not limited by stationary monitor density. While it also has significant limitations, it may provide valuable information to guide risk assessment and inform public health policy in vulnerable populations [25,26].

Our goal was to develop easily replicable, low-cost mechanisms to quantify exposure in high risk populations that can be replicated by public health agencies and advocacy groups. To test these techniques, in this study, we utilized local knowledge mapping techniques to generate “activity space” data for individuals experiencing homelessness. By combining this with validated, satellite-derived estimates of PM2.5, we aimed to quantify the exposure to ambient PM2.5 experienced by individuals living with unsheltered homelessness within a semi-rural county

## 2. Methods and Materials

Each year, communities across the U.S. participate in the Point in Time count (PIT) [27]. The PIT is designed to provide a census of people experiencing homelessness [27]. The data from this activity are then submitted to the Department of Housing and Urban Development (HUD) for federal funds to support services to this population. While the PIT is administered one night each January, there can also be related activities, termed magnet events, occurring during this time period to provide outreach. It is during such magnet events that this survey was conducted in collaboration with the Tulare California Health and Human Services Agency (HHS) during the 2019 calendar year. Specifically, three magnet events were held across incorporated areas of Tulare County. Tulare County is a semi-rural county located in inland California. The county has a population of 466,054 individuals, with three main population centers (Tulare, Visalia, and Porterville) [28]. Agriculture is the major economic driver [29]. Tulare County also has one of the highest percentages of individuals experiencing unsheltered chronic homelessness within the United States [30].

A table was set up at each of these events where intake personnel explained the purpose of the survey, how results would be used, described the compensation, and obtained consent. At the suggestion of local homeless service providers, participants were compensated with USD 5 gift cards for McDonalds or with bus passes. This study was approved by the Case Western Reserve University IRB (# 20191570). All individuals experiencing homelessness participating in PIT activities were eligible to participate.

After consent, participants were asked to mark on a printed base map places of importance to them, as well as describing when and why they used these spaces.

Completed maps were digitized so that participants’ marked locations could be placed in their real-world location in a GIS and the accompanying descriptions included in association with their geographic features. Self-reported demographic data, including gender identity and self-reported race, were collected as part of the broader PIT. Participants were afforded the opportunity to provide descriptions of why a space was perceived as “safe” or “unsafe”—these, along with their general comments about the mapping process, were recorded during data collection.

Activity spaces were derived for each participant, utilizing a combination of their self-reported day and night time locations, along with locations in which they obtained healthcare or services. These activity spaces were then overlaid with satellite-derived annual PM2.5 measurements at 0.01° × 0.01° (1 km) spatial resolution for the calendar year (2018) preceding activity space data collection. These estimates were derived by combining aerosol optical depth (AOD) measurements with the GEOS-Chem chemical transport model and calibrated using geographically weighted regression techniques [31]. For activity spaces smaller than 1 km, an additional 500 m buffer was created during analysis of annual PM2.5 concentrations to align the geographic units of measure. PM2.5 measurements at nocturnal “point” locations were estimated utilizing the same dataset.

Stationary emission sources were mapped utilizing the California Air Board stationary emissions inventory, and the average number of stationary emitters within each activity space was calculated [32]. Utilizing city limit data from the Tulare county resource management agency, density of emitters and estimated satellite-derived PM2.5 was calculated at the city level [32,33]. California air board data were utilized to map the location of stationary PM2.5 monitors within Tulare County. These monitors are both designed to function at the neighborhood scale, with an estimated spatial range of between 0.5 and 4 km [34]. Given this, 1 km buffers were generated around each monitor location, which were then utilized to calculate satellite-derived PM2.5 estimates over the location of optimal monitor performance.

Descriptive statistics were calculated utilizing R version 4.1.2 (R Foundation for Statistical Computing, Vienna, Austria).Wilcoxon signed-rank testing was utilized to compare median values for activity space size and emitter distribution. ANCOVA testing was utilized to compare mean PM2.5 concentrations across locations, as well as to assess for an interaction between participant-specific variables, mean PM2.5 exposure, and activity space size. Pearson’s chi-squared test was utilized to compare between group means. Significance was defined as a *p*-value less than 0.05.

## 3. Results

A total of 62 individuals experiencing homelessness participated in this study, including 17, 19, and 26 each at the Porterville, Tulare, and Visalia sites, respectively. Across all sites, the participants were racially and ethnically representative of their areas, with most being white and at least half self-identifying as Hispanic or Latino (Table 1). In addition, 58% identified as female and 42% identified as male. The majority (49%) were 45–64 years old, and most (69%) had resided in Tulare County for more than 10 years. This older and local population should be considered when interpreting results of this study. The median activity space size was 4.7 km^2^, although significant variation in activity space size was noted (IQR 1.6–10.5 km^2^) (Table 2). We did not detect any association between activity space size and participant self-reported gender or race.

Estimated PM2.5 varied at the city level, although this difference was not statistically significant. However, a significant degree of variation in estimated PM2.5 exposure was noted between individual participants, both when assessing utilizing activity space metrics and when utilizing single nocturnal point location estimates (Table 2). Variation in within-activity space and point level PM2.5 exposure was seen by township, with the highest activity space PM2.5 measurements noted in individuals experiencing homelessness residing in Visalia and Porterville (Table 2). Activity space size was not associated with a difference in PM2.5 exposure (*p* = 0.619).

**Figure 1:**
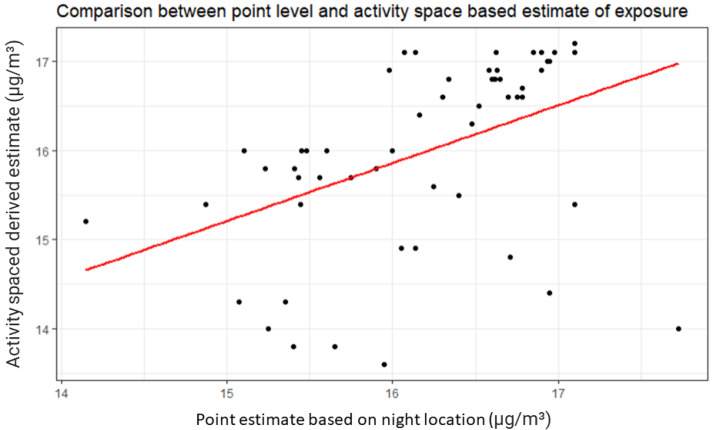
Comparison of individual point level and activity-space-derived estimates of annual mean PM2.5 exposure.

While a relationship was seen between point level and activity space level estimates of PM2.5 exposure, there was significant variation in estimated PM2.5 exposure with these different metrics at the participant level (Figure 1). Annual mean weighted PM2.5 as measured by the United States Environmental Protection Agency (US EPA) stationary monitors for Tulare County was 16.46 μg/m^3^ at the Porterville site and 17.49 μg/m^3^ at the Visalia site. By comparison, satellite-derived estimates of the mean PM2.5 concentration within a 1 km buffer of monitoring stations were 13.63 μg/m^3^ and 16.7 μg/m^3^, respectively.

A total of 518 registered stationary emitters of both fine and coarse particulate matter were identified. While the number of emitters varied between cities, density of emitters did not vary significantly (Table 2). However, this measurement does not account for clustering of emitters in urban areas, as evidenced by the fact that significant variation was seen in the number of emitters located within a participant activity space, with a median of six emitters per space (IQR 2–17). Estimated particulate matter release by emitters within activity spaces also varied, with a median estimated total particulate matter emission of 0.37 tons/year (IQR 0.005–2.39 tons). A statistically significant relationship between activity space size and number of emitters within the activity space was detected (*p* < 0.001). However, a relationship between activity-space-specific PM2.5 exposure and emitter density was not detected.

## 4. Discussion

A significant difference in exposure to ambient PM2.5 was detected between townships within Tulare County. This difference was not captured by existing stationary monitor data. All participants within our sample experienced estimated PM2.5 exposure in excess of the US EPA primary and secondary National Air Quality Standard of 12 μg/m^3^ and 15 μg/m^3^, respectively [35]. Exposure to ambient particulate matter in excess of the National Air Quality Standard has been linked with an increased risk of premature mortality and cardiovascular disease at a population level. Variation in estimated exposure was seen between participants. Given that even small differences in exposure to PM2.5 have been linked with differences in health outcomes at the population level, identifying this hyperlocal and local variation in PM2.5 may have significant health implications.

At the participant level, activity-space-derived estimates of PM2.5 differed from those derived from estimation utilizing a single point measurement alone. This variation reflects the potential limitations of much of the existing air pollution exposure literature, which relies on an assumption of stationarity. Significant variation in activity space size was seen across all three townships within our county. Some participants reported spending the vast majority of their day and night in the same location without moving. Others utilized multiple day and night locations, with a maximum reported activity space of 26.25 km. Reliance on exposure assessment based on a single site may result in misclassification of exposure. This has significant implications for research, which aims to link air pollution exposure to health impacts, particularly when sample sizes are low. A significant relationship between median activity space size and average PM2.5 exposure was not detected within our sample. This may reflect the high levels of PM2.5 exposure faced by our sample as a whole. Regardless, the variation in mobility between participants highlights the need to move beyond residential-address-based assessment of exposure.

Within our cohort, the vast majority of our participants were exposed to at least one stationary emitter, with many activity spaces enclosing multiple stationary emission sources of particulate matter. While activity space size did correlate with the number of emitters within the activity space, a relationship between emitter density and mean activity space PM2.5 concentrations was not detected. This reflects the fact that while satellite-derived data provide a novel mechanism for quantifying air pollution exposure at a more granular scale, this methodology still has the potential to under-estimate “hyperlocal” exposure to ambient air pollution. Similarly, our previous research found that individuals experiencing homelessness within this sample resided in close proximity to major roadways [17]. Traffic-related air pollution has been shown to display spatial variation at the 100–200 m range, which is unlikely to be captured even at the spatial granularity provided by satellite estimates [23,36]. Enhanced, multi-modal approaches to quantify air pollution exposure are still needed, particularly in neighborhoods and communities that may bear a disproportionate burden of air pollution exposure.

While the variation detected in mean PM2.5 exposure within our study population was small, increasing data suggests that even small variations in PM2.5 may be clinically significant. Even at concentrations below the national air quality standard, PM2.5 exposure is associated with an increased risk of cardiovascular and all-cause mortality [37]. Variation in PM2.5 exposure as small and as low as 1 μg/m^3^ has been associated with a significant increase in mortality across multiple cohorts [38,39]. Individuals experiencing unsheltered homelessness are already recognized to face an increased risk of chronic respiratory and cardiac disease [9,14]. Rates of tobacco use are also high [40]. In this setting, the health impacts of exposure to PM2.5 and other ambient air pollution may be further amplified.

Our study has several limitations. Most importantly, while satellite-derived estimates allow for an increased granularity of exposure assessment compared with the existing stationary monitor data within Tulare County, our findings suggest that satellite-derived measurements may still not adequately capture patterns of “hyperlocal” exposure. Additionally, our study did not attempt to correlate exposure to ambient air pollution with respiratory symptoms. Further longitudinal research quantifying the traffic-related air pollution associated with experiencing unsheltered homelessness and the health impacts associated with these exposures is needed to support public health interventions in this vulnerable population. Combining the techniques highlighted in our paper with technology such as low-cost stationary monitors may allow for targeted hot spot monitoring, particularly at high-risk locations such as near-roadway encampments.

Importantly, this study did not attempt to compare the exposure to air pollution faced by individuals experiencing homelessness with the exposure faced by other high-risk groups within the county. Those residing near major traffic thoroughfares, and in close proximity to stationary emitters, may also bear a disproportionate burden of air pollution exposure. More work is needed to further characterize the range of risk factors associated with residential air pollution exposure.

Disparities in air pollution exposure are increasingly recognized [2,3]. In qualitative interviews conducted during this study period, Black and female individuals experiencing homelessness reported residing closer to heavily populated commercial areas and avoiding parks and green-space due to concerns for safety. Given this, we hypothesize that due to the impacts of sexism and racism, a relationship between race, gender, and PM2.5 exposure could exist. Our study was under-powered to explore this relationship; however, concern remains that racism and sexism may further exacerbate the impact of unsheltered homelessness on an individual’s risk of exposure. Recognizing the potential interplay between discrimination and air pollution exposure, more work is needed to explore this relationship, both among individuals experiencing homelessness and across other vulnerable populations at risk of air pollution exposure.

## 5. Conclusions

Satellite-derived estimates of air pollution exposure provide a novel mechanism to quantify exposure to air pollution faced by individuals experiencing homelessness. Particularly in regions where air pollution monitoring is limited, the use of satellite-derived estimates along with other measures of air pollution exposure may facilitate identification of air pollution “hot spots”, guiding public health advocacy and intervention. With the increasing interest in citizen science-based initiatives to quantify and improve air pollution exposure within vulnerable communities, local knowledge mapping represents a cost-effective, easily replicable tool to enrich data collection and improve exposure assessment. When combined with local spatial data, these techniques have the potential to guide both further monitoring and local level policy interventions, improving the health of neighborhoods and communities where exposure is disproportionate.

## Figures and Tables

**Table 1:** Description of participants by location of local knowledge mapping completion.

	Porterville	Tulare	Visalia	Total	*%*
**PARTICIPANTS (*n*)**	17	19	26	62	
**RESIDENCE (years)**					
**<1**	2	0	4	6	10
**1–3**	1	1	2	4	6
**4–6**	0	1	5	6	10
**7–9**	3	1	0	4	6
**>10**	11	16	15	42	68
**AGE (years)**					
**18–29**	2	1	4	7	11
**30–44**	9	5	7	21	34
**45–64**	4	13	13	30	48
**>65**	1	0	2	3	5
**GENDER IDENTITY**					
**Male**	3	12	11	26	42
**Female**	14	7	15	36	58
**RACE AND ETHNICITY**					
**White/Caucasian**	12	13	10	35	56
**Black/African American**	0	1	3	4	6
**Asian/Pacific Islander**	0	0	0	0	0
**Native American/American Indian**	3	0	1	4	6
**Other**	2	5	9	16	26
**Hispanic/Latino**	6	6	15	27	44

**Table 2:** Distribution of activity space size and fine particulate matter (PM2.5) concentration by township within Tulare County. SD—standard deviation; IQR—interquartile range.

Variable	Visalia	Tulare	Porterville	*p*-Value
**Annual mean PM2.5 (μg/m^3^) (SD)**	15.6 (1.1)	14.17 (1.36)	15.3 (1.63)	0.98
**Emitter count (*n*)**	203	102	73	<0.001
**Emitter density (emitters/m^3^)**	5.59	4.85	4.12	0.89
**Activity space-specific mean annual PM2.5 (μg/m^3^) (SD)**	16.64 (0.27)	15.39 (0.41)	16.52 (0.62)	<0.001
**Nocturnal location mean annual PM2.5 (μg/m^3^)(SD)**	16.05 (1.27)	15.52 (0.63)	16.33 (1.0)	0.04
**Median emitters per activity space (emitters/m^3^) (IQR)**	11 (4–18)	11 (2–14)	3 (0–10)	0.07
**Median activity space size (km)(IQR)**	6.96 (3.26–11.5)	5.04 (1.02–9.43)	3.02 (0.01–5.74)	0.07

## Data Availability

The satellite derived PM2.5 estimates referenced in this paper can be accessed at https://sites.wustl.edu/acag/datasets/surface-pm2-5/ (accessed on 15 October 2021).
